# Temperature-Dependent Circularly Polarized Luminescence Measurement Using KBr Pellet Method

**DOI:** 10.3389/fchem.2020.00527

**Published:** 2020-06-23

**Authors:** Yoshiro Kondo, Satoko Suzuki, Masayuki Watanabe, Akio Kaneta, Paolo Albertini, Koushi Nagamori

**Affiliations:** ^1^JASCO Corporation, Hachioji, Japan; ^2^JASCO Europe srl, Cremella, Italy

**Keywords:** CPL, europium complex, solid-state CPL measurement, temperature-dependent CPL measurements, KBr pellet

## Abstract

Circularly polarized luminescence (CPL) spectroscopy measures the difference in luminescence intensity between left- and right-circularly polarized light, and is often used to analyze the structure of chiral molecules in their excited state. Recently, it has found an increasing range of applications in the analysis of molecules that emit circularly polarized light and can be employed in 3D displays. Thus, the number of articles focusing on CPL spectroscopy has increased dramatically. However, since the luminescence dissymmetry factor (*g*_lum_) for organic compounds is generally <|0.01|, CPL spectrometers must offer high sensitivity and produce spectra that are artifact-free for chiral molecules. Until now, the principal targets of CPL measurements have been solution samples. However, for practical device applications, it is also necessary to be able to measure the CPL spectra of solid-state samples. In addition, since electronic devices often operate at high temperatures, it is important to evaluate the thermal dependence of the CPL characteristics. Moreover, in the measurement of solid-state samples, the degree of anisotropy of the samples must be evaluated, because a large degree of anisotropy can cause artifacts. Therefore, we describe methods to evaluate the degree of anisotropy of solid-state samples and their high-temperature applications.

## Introduction

Recently, circularly polarized luminescence (CPL) spectroscopy has attracted attention in the study of optically active substances. Circular dichroism (CD) spectroscopy is used for structural analysis of the ground state of such substances, while CPL spectroscopy is a complementarily method that can obtain information about the excited state.

The CPL signal is defined as the difference in luminescence intensity between left- and right-circularly polarized light, and the luminescence dissymmetry factor (*g*_lum_) is defined as:

$$\begin{array}{l} g_{lum}=\frac{2\left(I_{L}-I_{R}\right)}{\left(I_{L}+I_{R}\right)}\;, \end{array}$$

where *I*_*L*_ and *I*_R_ are the luminescence intensities of left- and right-hand circularly polarized light, respectively. *g*_lum_ takes a value from −2 to +2, and is +2 for only left circularly polarized light, and −2 for only right circularly polarized light. The value measured by a CPL spectrometer is often output in the form of Δ*I* or θ (ellipticity in millidegrees). In a JASCO CPL-300 CPL spectrometer using lock-in detection, calibration is performed so that the following relationship is maintained between θ and *g*_lum_:

$$\begin{array}{l} g_{lum}=\frac{6.9813\times 10^{-5}\times \theta }{I} \end{array}$$

where *I* is the total luminescence intensity measured by the CPL spectrometer at the same time as the CPL spectrum.

In recent years, organic compounds exhibiting very large *g*_lum_ values have been synthesized. Sato et al. (2017) reported *g*_lum_ = ±0.152 for cylindrical organic molecules, and Shen et al. (2015) reported *g*_lum_ = −2.3 × 10^−2^ for supramolecular gels. However, for most organic compounds, *g*_lum_ is <|0.01|. Also, in the field of biomolecules, CPL of green fluorescent protein has been reported, but the *g*_lum_ value of 1.8 × 10^−3^ is small (Goto et al., 2010). In addition, in lanthanoid complexes, the CPL signal associated with f → f transitions has a large *g*_lum_, but the CPL band is very sharp (Brittain and Richardson, 1976; Zinna and Bari, 2015; Zinna et al., 2015; Hasegawa et al., 2020).

Until now, the principal targets of CPL measurements have been solution samples. However, in recent years, CPL measurements of solid-state samples have attracted attention. Nakabayashi et al. (2014) reported a peak shift in the fluorescence spectrum and inversion of the sign in the CPL spectrum when binaphthyl-crown ether-pyrene was dispersed in a chloroform solution or a PMMA film. Kimoto et al. (2013) reported that the sign of the CPL spectrum of binaphthyl fluorophores was reversed in a KBr pellet and a PMMA film. This also suggests that it is possible to control the CPL properties by changing the environment of the chiral compound without using an enantiomer. Taniguchi et al. (2019) reported the observation of aggregation-induced enhanced (AIEnh) CPL of chiral perylene diimide fluorophores in a KBr pellet, a PMMA film and a *myo*-IPU-film. Louis et al. (2019) reported mechano-responsive CPL by thermal annealing and smearing of a solid-state sample.

Compounds that emit circularly polarized light have potential applications to optical devices such as displays (Brandt et al., 2016). However, in order to achieve this, it is necessary to be able to perform solid-state CPL measurements. In addition, since electronic devices often operate at high temperatures, it is important to evaluate the temperature dependence of the CPL characteristics. Although there have been some reports on solid-state CPL measurements of samples in KBr pellets (Nishiguchi et al., 2011; Taniguchi et al., 2015) and PMMA films (Kimoto et al., 2012, 2013; Nakabayashi et al., 2014), the number of studies is still quite small. Okazaki et al. (2016) and Kumar et al. (2014) studied the temperature dependence of CPL characteristics, but again these reports are limited. Moreover, in the measurement of solid-state samples, the degree of anisotropy of the samples must be evaluated, because a large degree of anisotropy can lead to artifacts. Anisotropic samples such as single crystals and oriented liquid crystals have a large degree of anisotropy and cannot be measured by conventional CPL spectrometers. However, solid samples such as those dispersed in KBr pellets, which do not exhibit anisotropy, can be measured by conventional CPL spectrometers. Here, we describe methods to evaluate the degree of anisotropy of solid-state samples, and high-temperature measurement techniques for such samples.

## Artifacts in CPL Measurements

Conventional CPL spectrometers employ a photoelastic modulator (PEM) and a lock-in amplifier. Normally, in lock-in detection, the CPL signal is detected at 50 kHz and the linearly polarized luminescence (LPL) signal is detected at 100 kHz. The 100 kHz signal is filtered by signal processing in the CPL spectrometer, but if it is too large, it cannot be completely removed, causing artifacts in the CPL spectrum. These artifacts cannot be ignored when measuring samples exhibiting large anisotropy such as single crystals or oriented liquid crystals. For the case of an ideal PEM and lock-in amplifier, only the 50 kHz signal will be detected. However, since this is impossible in reality, the degree of anisotropy must be evaluated. The following two cases can be considered for solid-state samples: (1) Highly oriented samples such as single crystals and liquid crystals, (2) samples in which molecules motion is restricted, but the molecules are randomly oriented, such as samples dispersed in KBr pellets, Nujol, and isotropic films.

In the case of (1), it is necessary to measure CPL spectra with a dedicated CPL spectrometer, for example using the method based on the Stokes-Mueller matrix analysis reported by Harada et al. (2012). In the case of (2), molecular motion is restricted, so that a LPL component due to fluorescence anisotropy may be detected. However, even using a conventional CPL spectrometer, artifacts due to fluorescence anisotropy can be eliminated using unpolarized incident light and 180° detection, or horizontally polarized incident light with 90° detection (Blok and Dekkers, 1990; Castiglioni et al., 2014).

The following methods can be used to determine whether a sample exhibits a large degree of anisotropy:

Before measuring CPL spectra, measure the LD spectrum of the sample and confirm that there is no signal associated with anisotropy.

Rotate the sample around the excitation light propagation axis and measure CPL spectra at different angles.

These methods are also effective for measuring CD spectra of solid samples (Castiglioni et al., 2009). If possible, it is desirable to measure enantiomers and confirm that the CD and CPL spectra exhibit mirror images.

## Principles of CPL Spectroscopy

In a CPL spectrometer, the beam from a light source is monochromated, and the sample is excited by unpolarized or linearly polarized light. The fluorescence in the left- and right-circularly polarized light emitted from the sample is then modulated into linearly polarized light at a frequency of 50 kHz using a PEM installed behind the sample. A polarizer behind the PEM passes either left- or right-circularly polarized fluorescent light in synchronization with the PEM modulation. This fluorescence is monochromated and then detected. The CPL signal is obtained by lock-in detection of the difference between the fluorescence intensities for the left- and right-circularly polarized light, again synchronized with the PEM modulation. The total fluorescence spectrum of the sample can be obtained simultaneously with the CPL spectrum.

A CPL spectrometer can use a laser, LED, or xenon lamp as a light source. A laser can emit a high-intensity light beam, and a LED has the advantages of being inexpensive and having a long life. On the other hand, a xenon lamp emits a large amount of energy in the ultraviolet to near-infrared region, and the optimal excitation wavelength for a given sample can be selected using a monochromator. Also, the use of an ozone-free xenon lamp eliminates the need for nitrogen purging. Since commercially available CPL spectrometers have become widespread, ozone-free xenon lamps are widely used.

The monochromator is generally either a diffraction grating or a prism. Although the former allows easy wavelength control, polarization effects due to Woods' anomalies and higher-order light may affect the CPL spectra. By using a prism, these problems can be eliminated.

Fluorescence in circularly polarized light from the sample is detected at 90 or 180° with respect to the excitation-side monochromator. The 90° arrangement has the advantage of being little affected by scattered excitation light, but in the case of solid or highly viscous samples, it is susceptible to artifacts unless the sample is irradiated with horizontally polarized incident light (Blok and Dekkers, 1990; Castiglioni et al., 2014). On the other hand, in the case of the 180° arrangement, the influence of artifacts can be greatly reduced, just by performing measurements with unpolarized excitation light. In this configuration, the influence of scattered excitation light and stray light can be reduced by employing a double-prism monochromator.

Various accessories are available for CPL spectrometers. To measure thin films or KBr pellets, a special holder adapted to the sample shape can be used. In addition, Peltier thermostatted cell holders can be used to control the sample temperature, and some can be used in both CPL and CD spectrometers. In a 180° CPL spectrometer, magnetic circularly polarized luminescence (MCPL) measurements can be performed by placing a magnet in the sample chamber to produce a magnetic field that is parallel to the light beam. MCPL spectroscopy provides information about the electronic structure of the excited states of molecules. Usually, a photomultiplier tube with high sensitivity in the ultraviolet to visible region is used as a detector in a CPL spectrometer. To measure NIR CPL spectra, an InGaAs detector can instead be used. To our knowledge, there have been no reports on CPL measurements in the NIR region, but this will hopefully change.

## Calibration of CPL Spectrometer

Wavelength calibration is indispensable in a CPL spectrometer. For an ultraviolet/visible spectrophotometer or a spectrofluorometer, calibration of the wavelength is generally performed using an emission line from a deuterium or mercury lamp. For example, the JASCO CPL-300 CPL spectrometer uses low-pressure mercury lamps for both the excitation and emission monochromators, and the wavelength can be calibrated.

Calibration of the CPL scale is essential for CPL spectrometers that use lock-in detection. One method of achieving this is to irradiate a sample solution with a known CD value at a certain wavelength using unpolarized light, and to measure the difference in intensity between the left- and right-circularly polarized transmitted light. Samples with known CD values include (1S)-(+)-10-camphorsulfonic acid (Schippers and Dekkers, 1981) and (1S)-(+)-10-camphorsulfonic acid ammonium salt (Takakuwa et al., 1985). Since the latter is not hygroscopic, it is useful for calibrating CPL spectrometers.

## CPL Measurements Using KBr Pellet Method

**Figure 1:**
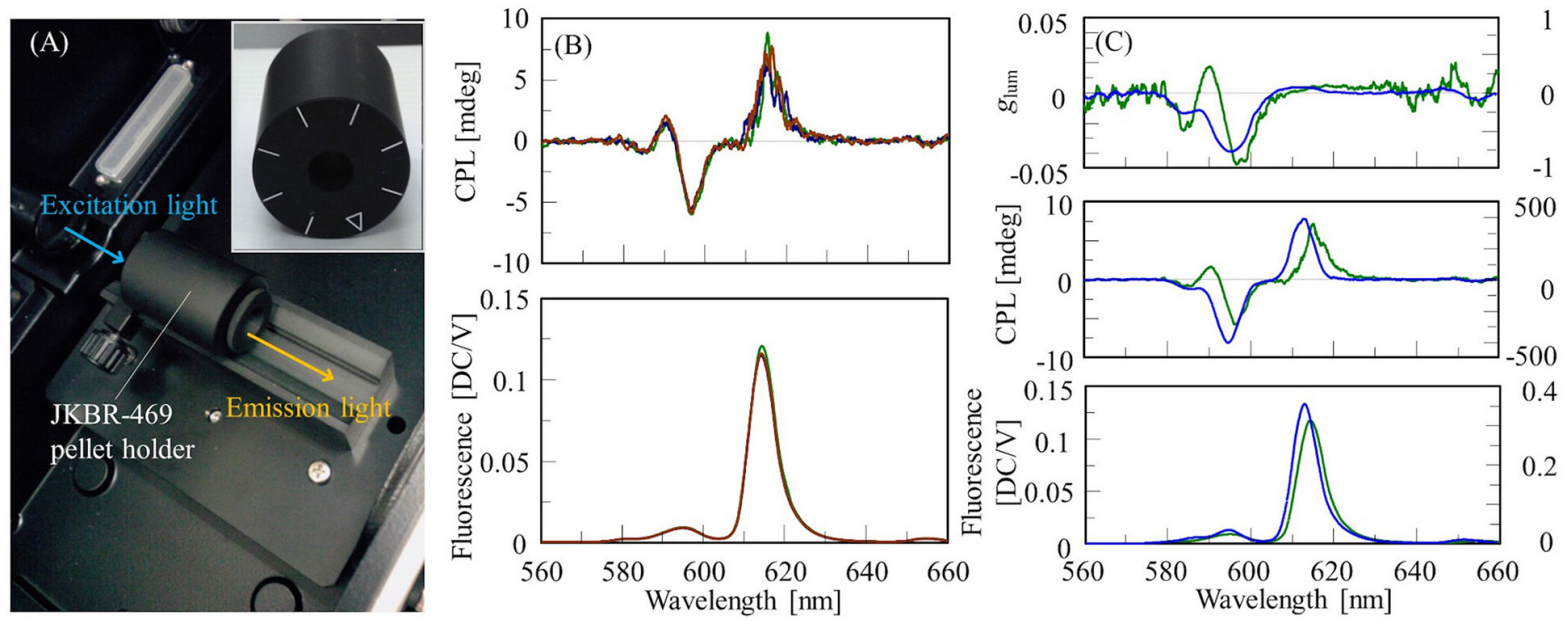
**(A)** Sample compartment of CPL-300 with installed JKBR-469 pellet holder, and front view of JKBR-469. **(B)** CPL and fluorescence spectra of Eu(facam)_3_/KBr pellet at rotation angles of 0° (green), 45° (blue), and 90° (red). **(C)** *g*_lum_, CPL and fluorescence spectra of Eu(facam)_3_/KBr pellet (green, left vertical axis) and in DMSO solution (blue, right vertical l axis).

To measure CPL spectra of solid-state samples using a conventional CPL spectrometer, available methods are the Nujol mull method, dispersing a sample in a film such as PMMA, and the KBr pellet method. In this section, we describe an example of measuring the CPL spectrum of a Eu(facam)_3_/KBr pellet. The pellet was prepared at a sample concentration of 5% (w/w) using the same procedure as that used for IR measurements, and had a diameter of 10 mm. The CPL spectrum was obtained using a JASCO CPL-300 CPL spectrometer. The KBr pellet was placed in a JKBR-469 pellet holder, and the fluorescence in the 180° direction was detected (Figure 1A). The excitation wavelength was 373 nm, and the emission bandwidth was 7 nm. The excitation light was unpolarized, thus suppressing CPL artifacts. Generally, to eliminate the possibility of artifacts being present, it is necessary to measure enantiomers and confirm that the CPL spectra are mirror images of each other. However, it is not always practical to obtain an enantiomer of a sample. In such a case, the presence of artifacts can be detected by rotating a solid-state sample like a polarizer with respect to the excitation light and measuring at different angles. If the CPL spectrum changes, artifacts are likely to be present. The JKBR-469 pellet holder has a mark every 45°, and by checking if the CPL spectrum changes by rotating the sample, the presence of artifacts can be determined. In this study, the CPL spectrum of the Eu(facam)_3_/KBr pellet was measured at 0°, 45°, and 90°, and no change was detected (Figure 1B).

CPL and fluorescence spectra of the Eu(facam)_3_/KBr pellet and the DMSO solution are shown in Figure 1C. The concentration of the Eu(facam)_3_/DMSO solution was 5 mg/mL, and the CPL spectra of Eu(facam)_3_/DMSO and Eu(facam)_3_/KBr were measured under the same conditions except for the number of accumulations. It is known that *g*_lum_ for Eu(facam)_3_ changes dramatically depending on the solvent used (Brittain and Richardson, 1976). In the present study, the spectrum of Eu(facam)_3_/KBr was different to that of Eu(facam)_3_/DMSO. For the KBr pellet, *g*_lum_ was −0.048 at 597 nm, which is about 16 times smaller than the value of −0.777 at 596 nm for the DMSO solution.

## Temperature-Dependent CPL Measurements Using KBr Pellet Method

**Figure 2:**
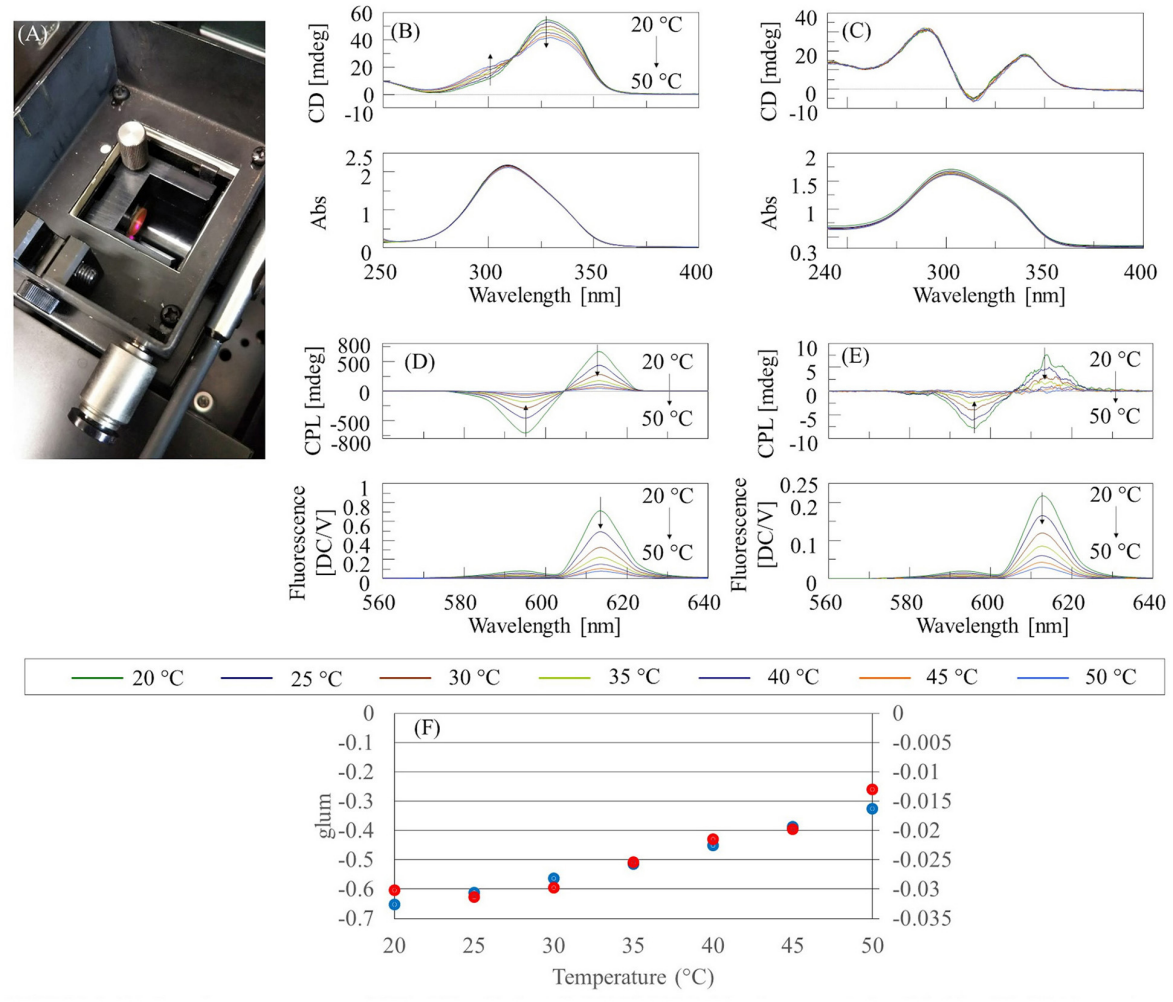
**(A)** Sample compartment of CPL-300 with installed PTC-510 Peltier thermostatted cell holder **(B)** CD/absorption spectra of Eu(facam)_3_/DMSO, **(C)** CD/absorption spectra of Eu(facam)_3_/KBr pellet, **(D)** CPL/fluorescence spectra of Eu(facam)_3_/DMSO, **(E)** CPL/fluorescence spectra of Eu(facam)_3_/KBr pellet, and **(F)** *g*_lum_ for DMSO solution (blue) and KBr pellet (red) at 596 nm.

The temperature dependence of the CPL and CD spectra of Eu(facam)_3_/KBr and Eu(facam)_3_/DMSO was investigated using a CPL-300 CPL spectrometer and a J-1500 CD spectrometer, respectively, together with a PTC-510 Peltier thermostatted cell holder (JASCO Corporation). The concentration of the Eu(facam)_3_/DMSO solution was 5 mg/mL, and the optical path length was 0.1 mm for CD measurements, and 10 mm for CPL measurements. The diameter of the KBr pellet was 10 mm. The concentration of Eu(facam)_3_ in the KBr pellet was 0.074% (w/w) for CD measurements, and 5% (w/w) for CPL measurements. The Eu(facam)_3_/KBr pellets were mounted on the handmade holder of the PTC-510 (Figure 2A).

The temperature-dependent CD and absorption spectra of Eu(facam)_3_/DMSO and Eu(facam)_3_/KBr are shown in Figures 2B,C, respectively. It can be seen that the CD spectrum of Eu(facam)_3_/DMSO changes with increasing temperature. On the other hand, for Eu(facam)_3_/KBr, there is almost no change in the CD spectrum with increasing temperature. This indicates that the structure of Eu(facam)_3_ in the ground state is more stable in the KBr pellet. No change is observed in the absorption spectrum of either Eu(facam)_3_/DMSO or Eu(facam)_3_/KBr. The temperature dependence of the CPL and fluorescence spectra of Eu(facam)_3_/DMSO and Eu(facam)_3_/KBr are shown in Figures 2D,E, respectively. For both samples, the CPL and fluorescence intensity decreases with increasing temperature. For Eu(facam)_3_/DMSO, |*g*_lum_| decreases monotonically with increasing temperature, whereas for Eu(facam)_3_/KBr, it remains almost constant up to 30°C and then decreases (Figure 2F). The different shapes of the CPL spectra in Figures 1C, 2E are due to the different emission bandwidths. When the Peltier thermostatted cell holder was used, the fluorescence intensity became weak, and so it was necessary to widen the bandwidth on the emission side. However, there was no problem in evaluating the relative change of *g*_lum_ with changing temperature.

## Future Prospects

Recently, there have been reports on the design and synthesis of chiral molecules based on theoretical strategies (Tanaka et al., 2018), and such studies are expected to become more common in the future. Accordingly, the importance of CPL spectroscopy is expected to increase. The targets of CPL spectroscopy are expanding from conventional solution samples to solid-state samples and temperature-dependent samples. We hope that this report will help further the development of research using CPL spectroscopy.

## Data Availability Statement

All datasets generated for this study are included in the article/supplementary material.

## Author Contributions

YK conceived and wrote the article. MW and AK developed the CPL spectrometer. SS, PA, and KN edited the article. All authors contributed to the article and approved the submitted version.

## Conflict of Interest

YK, SS, MW, AK, and KN were employed by the company JASCO Corporation. PA was employed by the company JASCO Europe srl.

## References

[B1] Blok P. M. L. Dekkers H. P. J. M. (1990). Measurement of the circular polarization of the luminescence of photoselected samples under artifact-free conditions. Appl. Spectrosc. 44, 305–309.

[B2] Brandt J. R. Wang X. Yang Y. Campbell A. J. Fuchter M. J. (2016). Circularly polarized phosphorescent electroluminescence with a high dissymmetry factor from PHOLEDs based on a platinahelicene. J. Am. Chem. Soc. 138, 9743–9746. 10.1021/jacs.6b02463 27434383

[B3] Brittain H. G. Richardson F. S. (1976). Circular polarized emission studies on the chiral nuclear magnetic resonance lanthanide shift reagent Tris(3-trifluoroacetyl-*d-*camphorato)europium(III). J. Am. Chem. Soc. 98, 5858–5863.

[B4] Castiglioni E. Abbate S. Lebon F. Longhi G. (2014). Chiroptical spectroscopic techniques based on fluorescence. Method Appl. Fluoresc. 2:024006. 10.1088/2050-6120/2/2/024006 29148463

[B5] Castiglioni E. Biscarini P. Abbate S. (2009). Experimental aspects of solid state circular dichroism. Chirality 21, 28–36. 10.1002/chir.20770 19722271

[B6] Goto H. Sawada I. Nomura N. (2010). Circular dichroism and circular polarized luminescence from a green fluorescent protein – Initial research for chiroptical emission of biological materials. Int. J. Polym. Mater. 59, 786–792. 10.1080/00914037.2010.483218

[B7] Harada T. Kuroda R. Moriyama H. (2012). Solid-state circularly polarized luminescence measurements: theoretical analysis. Chem. Phys. Lett. 530, 126–131. 10.1016/j.cplett.2012.01.059

[B8] Hasegawa M. Iwasawa D. Kawaguchi T. Koike H. Saso A. Ogata S.. (2020). Chiroptical spectroscopic studies on lanthanide complexes with valinamide derivatives in solution. ChemPlusChem. 85, 294–300. 10.1002/cplu.201900692 31967409

[B9] Kimoto T. Amako T. Tajima N. Kuroda R. Fujiki M. Imai Y. (2013). Control of solid-state circularly polarized luminescence of binaphthyl organic fluorophores through environmental changes. Asian J. Org. Chem. 2, 404–410. 10.1002/ajos.20130034

[B10] Kimoto T. Tajima N. Fujiki M. Imai Y. (2012). Control of circularly polarized luminescence by using open-and closed-type binaphthyl derivatives with the same axial chirality. Chem. Asian. J. 7, 2836–2841. 10.1002/asia.201200725 23038101

[B11] Kumar J. Nakashima T. Tsumatori H. Kawai T. (2014). Circularly polarized luminescence in chiral aggregates: Dependence of morphology on luminescence dissymmetry. J. Phys. Chem. Lett. 5, 316–321. 10.1021/jz402615n 26270706

[B12] Louis M. Sethy R. Kumar J. Katao S. Guillot R. Nakashima T.. (2019). Mechano-responsive circularly polarized luminescence of organic solid-state chiral emitters. Chem. Sci., 10, 843–847. 10.1039/c8sc04026e 30774879 PMC6345345

[B13] Nakabayashi K. Amako T. Tajima N. Fujiki M. Imai Y. (2014). Nonclassical dual control of circularly polarized luminescence modes of binaphthyl-pyrene organic fluorophores in fluidic and glassy media. Chem. Commun. 50, 13228–13230. 10.1039/c4cc02946a 24934379

[B14] Nishiguchi N. Kinuta T. Nakano Y. Harada T. Tajima N. Sato T.. (2011). Control of the solid-state chiral optical properties of a supramolecular organic fluorophore containing 4-(2-arylethynyl)-benzoic acid. Chem. Asian. J. 6, 1092–1098. 10.1002/asia.201000780 21265024

[B15] Okazaki Y. Goto T. Sakaguchi R. Kuwahara Y. Takafuji M. Oda R. (2016). Facile and versatile approach for generating circularly polarized luminescence by non-chiral, low-molecular dye-on-nanotemplate composite system. Chem. Lett. 45, 448–450. 10.1246/cl.060041

[B16] Sato S. Yoshii A. Takahashi S. Furumi S. Takeuchi M. Isobe H. (2017). Chiral intertwined spirals and magnetic transition dipole moments dictated by cylinder helicity. Proc. Natl. Acad. Sci. U.S.A. 114, 13097–13101. 10.1073/pnas.1717524114 29180419 PMC5740620

[B17] Schippers P. H. Dekkers H. P. M. J. (1981). Direct determination of absolute circular dichroism data and calibration of commercial instruments, Anal. Chem. 53, 778–782.

[B18] Shen Z. Wang T. Shi L. Tang Z. Liu M. (2015). Strong circularly polarized luminescence from the supramolecular gels of an achiral gelator: tunable intensity and handedness. Chem. Sci. 6, 4264–4272. 10.1039/c5sc01056j 29218194 PMC5707475

[B19] Takakuwa T. Konno T. Meguro H. (1985). A new standard substance for calibration of circular dichroism: ammonium d-10-camphorsulfonate. Anal. Sci. 1, 215–218.

[B20] Tanaka H. Ikenosako M. Kato Y. Fujiki M. Inoue Y. Mori T. (2018). Symmetry-based rational design for boosting chiroptical responses. *Commun*. Chem. 1, 1–8. 10.1038/s42004-018-0035-x

[B21] Taniguchi A. Kaji D. Hara N. Murata R. Akiyama S. Harada T. (2019). Solid-state AIEnh-circularly polarized luminescence of chiral perylene diimide fluorophores. RSC Adv. 9, 1976–1981. 10.1039/c8ra09785b PMC9059715 35516153

[B22] Taniguchi N. Nakabayashi K. Harada T. Tajima N. Shizuma M. Fujiki M. (2015). Circularly polarized luminescence of chiral binaphthyl with achiral terthiophene fluorophores. Chem. Lett. 44, 598–600. 10.1246/cl.150011

[B23] Zinna F. Bari L. D. (2015). Lanthanide circularly polarized luminescence: bases and applications. Chirality 27, 1–13. 10.1002/chir.22382 25318867

[B24] Zinna F. Resta C. Abbate S. Castiglioni E. Longhi G. Mineo P.. (2015). Circularly polarized luminescence under near-UV excitation and structural elucidation of a Eu complex. Chem. Commun. 51, 11903–11906. 10.1039/c5cc04283f 26112132

